# Chinese Herbal Medicine for Postinfectious Cough: A Systematic Review of Randomized Controlled Trials

**DOI:** 10.1155/2013/906765

**Published:** 2013-11-20

**Authors:** Wei Liu, Hong-Li Jiang, Bing Mao

**Affiliations:** Department of Integrated Traditional Chinese and Western Medicine, West China Hospital, West China School of Clinical Medicine, Sichuan University, Chengdu 610041, China

## Abstract

Chinese herbal medicine has been commonly used in the treatment of postinfectious cough. The aim of this review is to systematically evaluate the efficacy and safety of Chinese herbal medicine for postinfectious cough. An extensive search for RCTs was performed using multiple electronic databases, supplemented with a manual search. All studies included were confirmed with specific inclusion criteria. Methodological quality of each study was examined according to the Cochrane risk of bias assessment. Quality of evidence was evaluated using rating approach developed by GRADE working group. The literature search yielded 352 results, of which 12 RCTs satisfied the inclusion criteria, offering moderate-to-high levels of evidence. Methodological quality was considered high in three trials, while in the other nine studies the unclear risk of bias was in the majority. Findings suggested that, compared with western conventional medicine or placebo, Chinese herbal medicine could effectively improve core symptoms of postinfectious cough, act better and have earlier antitussive effect, and enhance patients' quality of life. No serious adverse event was reported.

## 1. Introduction

Patients who complain of a persistent cough lasting >3 weeks but not >8 weeks after experiencing the acute symptoms of an upper respiratory tract infection are considered to have a postinfectious cough (PIC) [1]. PIC is supposed to be the most common cause of subacute cough [2], which is distinguished from the chronic cough by the duration of coughing [3]. For adults, retrospective studies of unselected patients with a history of upper respiratory tract infection showed that the frequency of PIC ranged from 11 to 25% [4], which increased to the range from 25 to 50% during outbreaks of atypical pathogens infections [5, 6]. Respiratory viruses, *M. pneumoniae, Chlamydophila pneumonia*, and some specific bacteria have been implicated in the cause of PIC [7, 8]. The pathogenesis of the PIC has not been clearly recognized. It was frequently thought to be relevant to multiple factors involving disruption of epithelial integrity, widespread airway inflammation, and bronchial hyperresponsiveness [9–12]. 

Although PIC is self-limited and will usually resolve on its own in time, persistent cough always incurs much health troubles and economic miseries for patients and their surroundings. Thus, medication therapy is imperative sometimes. Up to now, optimal treatment of western conventional medication (WCM) for patients with PIC is not known [1]. Symptomatic therapeutic medications including antihistamine, decongestant, and ipratropium bromide are being commonly used. Besides, brief course of inhaled or oral corticosteroids [13] has sometimes been administered in view of airway inflammations. But corticosteroids were generally used just in patients with severe paroxysms of PIC for their remarkable side effects [1]. Central acting antitussive agent such as codeine or dextromethorphan would be considered, as a last resort, in those patients who are resistant to other treatment approaches; however, there have been no clinical trials conducted to support its effect [1]. In addition, antibiotics are usually abused in treatment of PIC. Therefore, research for optimal treatment of PIC is particularly needed [14, 15].

TCM has unique understanding of PIC and has established its own diagnosis and treatment approach. In TCM theory system, PIC, equivalent to the category of exogenous cough, is caused by invasion of external evil factors (wind-evil, cold-evil, summer-damp-evil, wet-evil, dryness-evil, and fire-evil). These factors disturb or suppress Qi activity in the lung, leading to acute cough. If things continue this way, the lung-Qi would be damaged and unable to drive evils out, leaving external evils lingering in the lung for a long time. In this case, patients would suffer a fairly long duration of cough. With thousands of years of experience, TCM clinicians summarized the most common pathomechanism of PIC as “disorder Qi activity in the lung” due to “wind-evil invading the lung.” The corresponding therapeutic method is “dispelling wind and dispersing the lung-Qi.” PIC is a disease with complex pathogenic conditions including phlegm turbidity and deficiency of the lung-Yin. So flexible treatment based on syndrome differentiation is important. 

 An increasing number of clinical trials on CHM for PIC have been performed. This current systematic review aims to collect the evidence from RCTs to evaluate the therapeutic effect and safety of CHM in the management of PIC.

## 2. Methods

### 2.1. Research Protocol

All methods were performed according to a predefined, unpublished protocol, which consisted of the search databases, detailed research question, search strategies, and eligibility criteria. The detailed research question included study design, patient characteristics, interventions, and outcomes. 

### 2.2. Database and Search Strategies

 Literature searches were conducted to identify reports of randomized controlled trials involving CHM for PIC in the following preliminary determined electronic databases: Chinese National Knowledge Infrastructure (CNKI), Wanfang Database, EMBASE, MEDLINE (PubMed), Cqvip Database, Google Scholar, Scholarly and Academic Information Navigator (CiNii), and Cochrane Library from inception to July 2013. Ongoing registered clinical trials were searched on the website of Chinese clinical trial registry (ChiCTR) (http://www.chictr.org/). Searches for relevant conference proceedings, unpublished literature, and studies (Table 1) included in previous relevant systematic reviews were performed. Moreover, manual searches for bibliographies of all retrieved literature sources were conducted for additional references. No language restriction was applied. 

The following phrases and their derivatives or relevant terms were utilized singly or in combination: “post-infectious cough,” “subacute cough,” “cough post influenza,” “postviral cough,” “post-cold cough,” “whooping cough,” “Chinese herbal medicine,” “traditional Chinese medicine,” and “randomized controlled trial.” The search terms were modified to adapt to different databases with a highly sensitive search strategy developed by the Cochrane Collaboration [16]. We contacted authors for further information or clarification. Searching work was done by two reviewers (Wei Liu and Hong-Li Jiang) independently. Searching results were cross-checked for accuracy.

### 2.3. Inclusion Criteria

#### 2.3.1. Types of Studies

All relevant randomized controlled trials or quasirandomized controlled trials that were published before July 20, 2013 were considered, regardless of blinding. 

#### 2.3.2. Types of Participants

Any patients with diagnosed and existing PIC, of either gender, any profession or ethnicity, and any ages ≥12, were included. Those without description of diagnostic criteria but stated patients with definite PIC were also considered. 

#### 2.3.3. Types of Interventions

Studies involving a comparison between CHM alone or in combination with WCM and the same WCM or placebo as controls were included. CHM included herbal extracted product, Chinese patent medicine (CPM), and self-modified herbal formula. CHM could be of any dose, duration, dosage form, and route of administration. Studies involving cointerventions of traditional extrapulmonary therapies such as acupuncture, cupping, or point application were excluded. 

#### 2.3.4. Outcomes

Primary outcomes measures were as follows: (1) cough symptom score, which consists of the daytime-score and the nighttime-score, ranging from 0 to 6 [17]; (2) cough relief time, defined as both the daytime-score and the nighttime-score ≤1, which lasted for 48 hours; (3) cough disappearance time, defined as both of the daytime-score and the nighttime-score = 0, which lasted for 48 hours. Secondary outcome measures were as follows: (1) Obvious effective rate, defined as reduced rate of symptoms score ≥70% according to the Guiding Principle of Clinical Research on New Drugs of TCM [18, 19]; (2) quality-of-life (QoL) score, evaluated using Cough-Specific Quality-of-Life Questionnaire (CQLQ) [20] or Leicester Cough Questionnaire (LCQ) [21]; (3) adverse events.

### 2.4. Studies Selection and Data Extraction

Two reviewers (Wei Liu and Hong-Li Jiang) independently screened the titles and abstracts of searching results against prespecified inclusion criteria to identify potential relevance that required full texts for further identification. Disagreements were resolved by consensus. All articles included were judged by the third reviewer (Bing Mao). 

Two reviewers (Wei Liu and Hong-Li Jiang) systematically extracted data regarding study design, demographic characteristics, interventions, and outcome measures independently. Discrepancies were resolved by discussion between the two reviewers or by consultation with the third arbitration (Bing Mao). 

### 2.5. Qualities Assessment

 We used risk of bias assessment tool recommended by the Cochrane Collaboration to address the following six domains: random sequence generation, allocation concealment, blinding of participants and personnel, blinding of outcome assessment, incomplete outcome data, selective outcome reporting, and “other bias” [22]. The risk of bias for each item was summarized as three levels: low, high, and unclear. The risk of bias graph was made using RevMan 5.2 software.

We also used GRADE approach to assess the quality of the evidence for each individual outcome. Besides within-study limitations of design and execution (methodological quality), the GRADE approach incorporates comprehensive considerations of the following four factors: directness of evidence, inconsistency of results, imprecision, and publication bias [23, 24]. Accordingly, we graded the quality of evidence as very low, low, moderate, or high. 

The first reviewer (Wei Liu) performed the quality assessments with supervision from the other two reviewers (Hong-Li Jiang and Bing Mao). 

### 2.6. Data Analysis

In this review, a formal meta-analysis would not be conducted for the predicted large heterogeneity across trials [25]. Therefore, a narrative synthesis approach was applied. 

## 3. Results

### 3.1. Description of Included Studies

**Figure 1:**
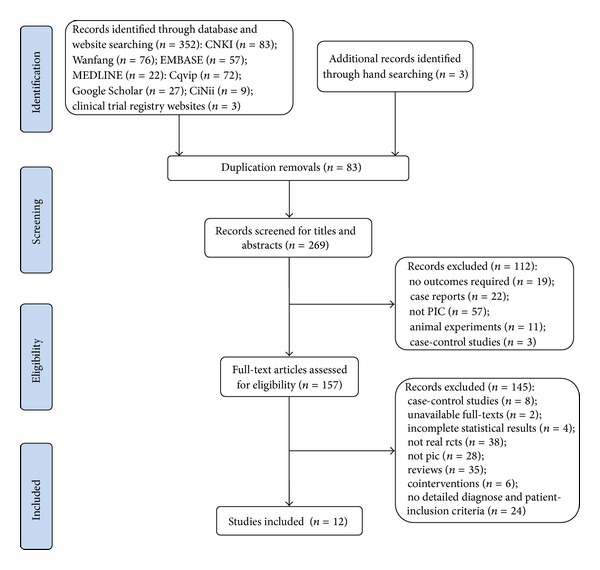
Process of study search and selection.

The search strategies came up with 352 potentially relevant citations. Twelve trials involving 1289 subjects satisfied all the inclusion criteria (Figure 1). One unpublished study [35] searched on the website of ChiCTR was included (registration number ChiCTR-TRC-12002297). Patients included in all studies were explicitly diagnosed as having PIC according to the nationwide unified western medicine diagnostic criteria [17] or ACCP Evidence-Based Clinical Practice Guidelines. TCM syndrome differentiation was identified based on the recognized guiding principles [18, 38]. TCM syndrome of each patient was specified in ten studies and it was defined as “syndrome of wind evil invading the lung” in seven studies. As to the interventions, two studies compared CHM with placebo [33, 35], five studies compared CHM with WCM [26, 29, 31, 34, 37], and two studies compared CHM adjuvantly used with WCM to the same WCM alone [27, 28], two studies compared two CPMs [30, 32]. Three trials reported a follow-up period to evaluate the sustained or subsequent effect of interventions [31, 33, 35]. CHMs used in the studies included were totally different. But nine of them [28–33, 35–37] were prescribed based on the same TCM therapeutic principle of “dispelling wind and dispersing the lung-Qi” (Table 2). 

### 3.2. Quality Assessment of Included Studies

In general, the unclear risk of bias is in the majority. Very limited information was available in many studies to permit a judgment of whether the risk of bias existed (Table 3). 

Appropriate random component in the sequence generation process was described in six trials [30–33, 35, 36]. One study [37] implemented a quasirandom method by allocating patients according to sequence number of visiting. In this situation, patients' assignments could possibly be foreseen, which would introduce high selection bias. 

Allocation concealment was presented in three trials [32, 33, 35]. One study [27] only pointed “envelopes” but did not specify whether they were sequentially numbered, properly opaque, or sealed. Other studies failed to show any information of allocation concealment. Thus, whether the randomization was effectively conducted was doubtful, leading to an unclear risk of selection bias.

Blinding was not addressed in most studies. Only three studies [32, 33, 35] claimed to be double-blind and elaborated the blinding method that was unlikely to be broken, contributing to a sufficient protection against bias. One study [37] stated single blind just in broad terms, so the performance risk of bias was classified as “unclear.” Three studies [27, 29, 34] were open-label trials, and the patients' knowledge of interventions they received was likely to result in a high risk of detection bias.

### 3.3. Quality of Evidence of Included Studies

The “GRADE profiler” of the Cochrane Collaboration Network was used to assess the individual outcome. The quality of evidence was labeled as moderate to high (Table 4).

### 3.4. Outcome Measures of Included Studies

Forest plots were used to show the statistical results of some outcome measures. Two studies [35, 36] divided patients into three groups, so each of them was regarded as two RCTs in the final analysis. 

#### 3.4.1. Cough Symptom Score

**Figure 2:**
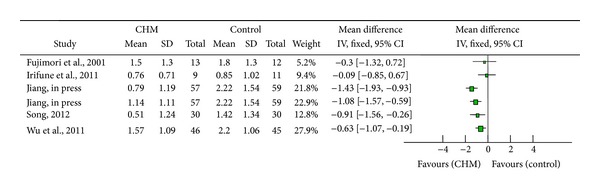
Cough symptom score analysis.

Cough symptom score was reported in five studies. Two studies [26, 27] showed no significant difference between bakumondo-to group and WCM group at the last visit of view, but both of them found that bakumondo-to group had a more quick antitussive effect. Three studies [30, 31, 35] concluded significant difference between the trial group and the control group (Figure 2).

#### 3.4.2. Cough Relief Time

**Figure 3:**
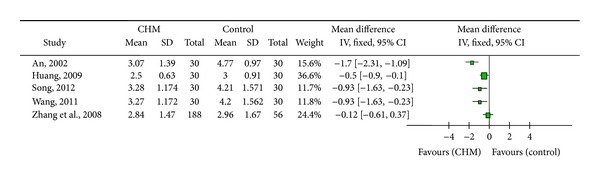
Cough relief time analysis.

Seven trials selected cough relief time as an outcome measure. For this indicator, six studies demonstrated statistically significant difference between the trial group and the control group [29, 30, 33–35, 37], indicating a more rapid cough relief effect of CHM, of which two studies [33, 35] estimated cough relief time for each group using Kaplan-Meier method (median cough relief time: T = 6 d, C = 7 d; log-rank test *P* = 0.026 [33]; median cough relief time: H = 4 d, L = 4 d, C = 6 d; log-rank test *P* < 0.001 [35]). In the other study [32], significant difference between two CPMs (Su-huang Zhi-ke capsule and Zhi-ke Ning-sou capsule) was not observed in statistical analysis (Figure 3).

#### 3.4.3. Cough Disappearance Time

 Cough disappearance time was investigated in two researches. One study [35] showed better effect favoring CHM groups and detected statistical significance on difference across three groups (median cough disappearance time: H = 8 d, L ≥ 10 d, C ≥ 10 d; log-rank test *P* < 0.001). The other study [32] demonstrated the same effect of two CPMs (Su-huang Zhi-ke capsule and Zhi-ke Ning-sou capsule) (MD = −0.11, 95% [0.83,0.61]). 

#### 3.4.4. Obvious Effective Rate

**Figure 4:**
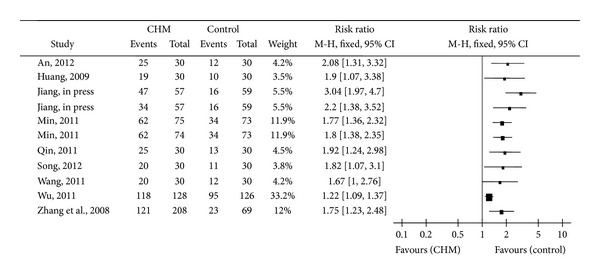
Obvious effective rate analysis.

Nine trials selected “effective rate” (ER) as an outcome measurement. In these studies, “rate ratio” (RR) was calculated as the ratio between the proportion of responders in the trial group and the proportion of responders in the control group. Responders were defined as those patients with an improvement rate of symptoms score ≥70%. For this indicator, eight studies were statistically significant, showing that CHM can improve clinical core symptoms of PIC [28, 30, 32–37]. The same effect of CHM and WCM was reported in one study [29] (Figure 4).

#### 3.4.5. Quality-of-Life Score

 Quality-of-life (QoL) evaluation was conducted in two studies. Both of them showed that CHM possessed a better effect in improving patients' quality of life. One study [35] calculated CQLQ total score and demonstrated significant difference across groups (*P* < 0.00001, MD = −8.34, 95% [−11.63, −5.05]). The other study [30] recorded physical, psychological, and social domains of LCQ, and difference of post treatment score for the three domains between a self-modified decoction (Qing-fei Zhi-ke decoction) and a CPM (Jizhi syrup) was statistically significant (MD and 95% for physical, psychological, and social domains, respectively: MD = −0.90, 95%[−1.01, −0.80]; MD = −3.00, 95%[−3.08, −2.92]; MD = −0.72, 95%[−0.87, −0.56]).

### 3.5. Adverse Events

**Figure 5:**
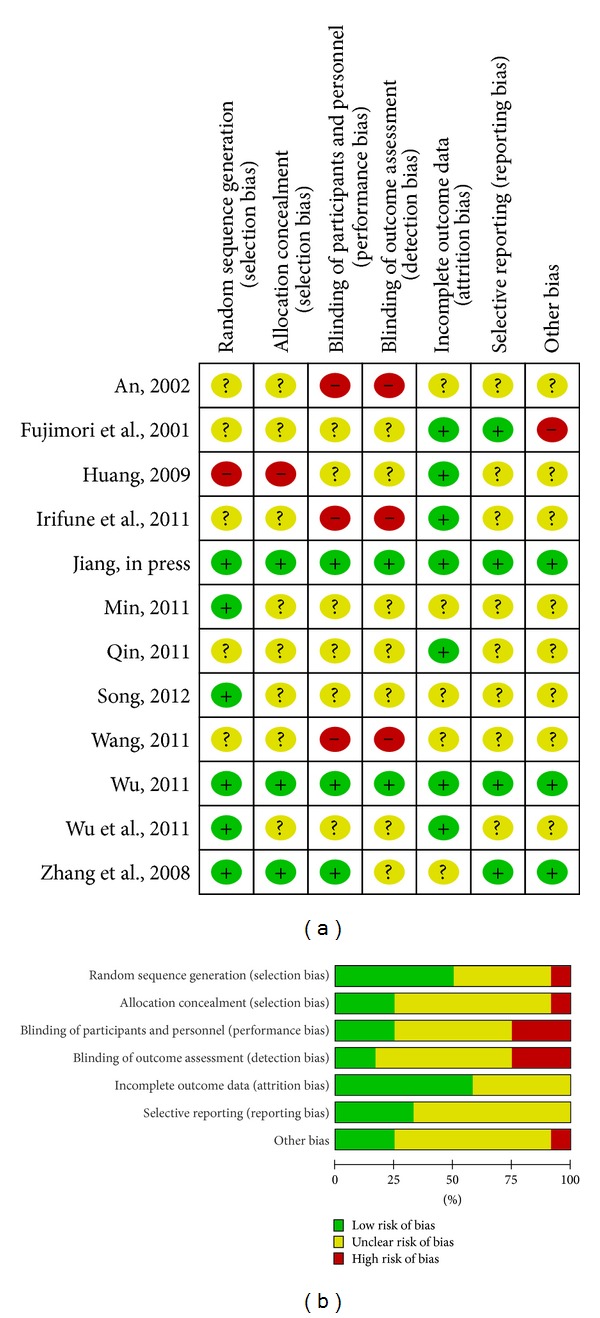
(a) Risk of bias summary: review authors' judgements about each risk of bias item for each included study, dash sign: high risk of bias, plus sign: low risk of bias, question mark sign: unclear risk of bias; (b) risk of bias graph: review of authors' judgements about each risk of bias item presented as percentages across all included studies.

In general, CHM was claimed to have fewer side effects compared with WCM. Adverse events were mentioned in eleven studies. Four studies [25, 28, 29, 33] reported that no adverse events were observed at the end of treatment. In seven studies [26, 30–32, 34–36], various adverse events were reported for 49 patients in the control group and for 114 patients in the trial group. All the adverse reactions were mild and did not affect results estimation (Figure 5).

## 4. Discussion

Broadly speaking, findings of this current review suggested that CHM may have potential positive clinical effect in the treatment of PIC, and the outcome evidence was relatively optimistic for us to make further research to draw a confirmative conclusion.

 Three included studies [32, 33, 35] were well-designed, leading to favorable methodological quality and robustness of results. Besides, reporting form and content of these three articles were comprehensive and they basically met the international criteria of CONSORT statement [39]. In other studies, limitations commonly concerned the issue of allocation concealment and blinding. Just like most of the TCM clinical trials, allocation concealment was not reported or not appropriately put into effect in most studies in this review. Only two studies [33, 35] were double-blind and placebo-controlled. Both of them explicitly claimed that outcome assessors and data analyzers were definitely unconscious of interventions that patients received. Placebo is considered as a welcome and reasonable control for effective estimation of CHM [40, 41], because CHM is considered to possess a potential psychological positive effect which is similar to placebo. So if intervention other than placebo is applied as control, the placebo-like effect of CHM may bring out false or exaggerated positive results in favor of the trial group. 

 Imperfections of included studies also lie in the common absence of sample size calculation. Only three studies [32, 33, 35] estimated sample size. Two studies [26, 27] recruited less than 30 cases in each group, so it was not ensured whether they could provide enough power to detect the difference between groups. In addition, a small sample also might cause exaggerated difference between groups. Therefore, outcomes in this case should be regarded with caution. 

Although the formulas used in the trials included in this review were not completely equivalent, nine of them [28–33, 35–37] were prescribed based on the same fundamental TCM therapeutic principle of “dispelling wind and dispersing the lung-Qi.” *Ephedra, Platycodon grandiflorus, Folium perilla, Almond, *and *Schizonepeta tenuifolia *were usually used in prescriptions with this function. The corresponding TCM syndrome of patients is “wind evil attacking the lung”, which is considered as the most common syndrome in patients with PIC. In addition, TCM therapeutic principles such as nourishing the lung-Yin, resolving phlegm, expelling cold, or eliminating heat were also commonly used for PIC.

Most studies implied good safety of CHM. However, some results should be treated with prudence because only three studies [27, 33, 35] judged the definite relevance between adverse events and corresponding interventions. As a significant complementary and alternative therapeutic method, TCM is traditionally regarded as natural with fewer side effects. Thus, some researchers tend to pay less attention to safety of CHM. But since the toxicological risks of CHM have been observed in many researches [42], more emphasis should be placed on this issue. 

In this review, only four studies [26, 27, 33, 35] declared ethical approval and only one study [35] completed clinical trial registration on a publicly accessible database before the trial was set about. Hence, compliance with ethical guideline and trial registration should be addressed in further researches to eventually strengthen the effectiveness and value of scientific evidence.

Although most of the studies in this review authoritatively recommended CHM for PIC, it is impossible to conduct a meaningful meta-analysis to prove the conclusion. Clinical investigators have to consider repeating studies with the same CHM for a powerfully statistical proved conclusion. Decoction, as a complex mixture, is usually prepared artificially. Therefore, the processes of decoction manufacturing and decoction administeration are unlikely to realize quality-and-quantity control. Accordingly, the consistency of decoction throughout the whole treatment course is suspected. In addition, if decoction is used as intervention in a research, blinding method is difficult to be implemented for it seems impossible to produce a simulation. Thus, prepared drugs formulated as granules, tables, pills, or capsules are in need to reduce bias in TCM clinical trials. 

## 5. Conclusion

Findings suggest that CHM may effectively improve core symptoms of PIC, act better and have earlier antitussive effect, and enhance patients' quality of life. CHM is relatively safe and well tolerated without serious side effects. The most common syndrome of patients with PIC is “syndrome of wind evil invading the lung”; correspondingly, “dispelling wind and dispersing the lung-Qi” is the commonest TCM therapeutic principle for PIC. Since some of the studies included in this review were well-designed and comprehensively reported, various limitations still existed. Therefore, confirmative conclusions are not allowed. But current evidence is promising for clinical investigators to do further in-depth researches. Larger-scale, multicentre, placebo-controlled studies for diverse populations are definitely welcomed. Action mechanism of CHM in treatment of PIC, which has been poorly known, needs to be researched to prove the effectiveness and safety of CHM in a more convincing and essential manner. 

## Figures and Tables

**Table 1:** Characteristics of the included studies.

Study ID	N* T/C	Number M/F	Age (mean ± SD or range, Y)	Study design	Interventions	Treating duration (D)	Outcomes	Dropout (T/C)	Adverse events (T/C)
Fujimori et al., 2001, Japan [26]	13/12	2/33	T: 31–81 C: 23–67	RCT	T: bakumondo-to extract granules; C: dextromethorphan	Seven	Cough symptom score	1 patient in the control group dropped out because of failure to bring a cough diary	No
Irifune et al., 2011, Japan [27]	9/11	8/12	T: 60.7 ± 12.7 C: 48.5 ± 19.8	Multicentre; RCT; open-label	T: procaterol hydrochloride (Meptin tablets) plus bakumondo-to extract granules (TJ-29); C: procaterol hydrochloride (Meptin tablets)	Fourteen	Cough symptom score	1 patient in the control group was excluded due to allocation error	Palpitation and hands tremor considered to be attributed to Meptin tablets were observed in 6 patients (4/2) Rash considered to be attributed to TJ-29 treatment was observed in 1 patient in the trial group
Qin, 2011, China [28]	30/30	31/29	T: 40.07 ± 14.14 C: 41.87 ± 13.57	RCT	T: Er-li decoction plus compound methoxyphenamine capsules; C: compound methoxyphenamine capsules	Ten	Obvious effective rate	No	NS
Wang, 2011, China [29]	30/30	33/27	T: 37.03 ± 9.946 C: 33.50 ± 9.662	RCT; open-label	T: Xuan-fei Zhi-sou decoction; C: pseudoephedrine hydrochloride, chlorpheniramine maleate, and dextromethorphan hydrobromide solution	Seven	Obvious effective rate; cough relief time	NS	No
Song, 2012, China [30]	30/30	19/41	T: 45.23 ± 11.25 C: 45.45 ± 10.52	RCT	T: Qing-fei Zhi-ke decoction; C: Jizhi syrup (CPM)	Fourteen	Cough symptom score; obvious effective rate; LCQ score; cough relief time	6 patients dropped out without explanations provided	No
Wu et al., 2011, China [31]	50/50	49/51	T: 19–64 C: 22–65	RCT	T: Shu-feng Xuan-fei decoction; C: chlorpheniramine maleate tablets plus pentoxyverine citrate tablets	Five	Cough symptom score	9 patients dropped out because of being lost to follow-up, poor compliance, and concomitantly taking other antitussive drugs (4/5)	Dry mouth was observed in 35 patients (7/28); dizziness was observed in 25 patients (1/24); drowsiness was observed in 14 patients in the control group; nausea was observed in 12 patients (2/9); constipation was observed in 9 patients (1/8)
Zhang et al., 2008, China [32]	208/69	114/163	T: 40.813 ± 12.024 C: 41.261 ± 11.843	Multicentre; RCT; DB; parallel-group	T: Su-huang Zhi-ke capsule (CPM); C: Zhi-ke Ning-sou capsule (CPM)	Seven	Obvious effective rate; cough relief time; cough disappearance time	6 patients dropped out without explanations provided (4/2)	Stomach upset was observed in 3 patients in the trial group
Wu, 2011, China [33]	143/134	122/155	T: 36.95 ± 12.67 C: 36.27 ± 11.42	Multicentre; RCT; DB; placebo-controlled; parallel-group	T: extract granules of Feng-han decoction or Feng-re decoction; C: placebo	Ten	Obvious effective rate; cough relief time	23 patients dropped out. Of which, 10 patients were lost to follow-up (6/4), 12 patients were required to withdraw (9/3), and 1 patient in the control group refused to take drug because of inefficacy	Adverse events without further specifications were observed in 37 patients (17/20)
An, 2002, China [34]	30/30	26/34	T: 37.53 ± 11.85 C: 34.50 ± 11.65	RCT; open-label	T: Zhi-ke Gu-biao decoction; C: pentoxyverine citrate tablets	Fourteen	Obvious effective rate; cough relief time	NS	No
Jiang, 2013, China [35]	57/57/59 (H/L/C)	72/101	H: 43.07 ± 12.67 L: 44.37 ± 12.38 C: 44.07 ± 12.65	Multicentre; RCT; DB; placebo-controlled; parallel-group	H: Qing-feng Gan-ke granules (CPM); L: Qing-feng Gan-ke granules; C: placebo	Fourteen	Obvious effective rate; cough relief time; cough disappearance time; CQLQ score; Cough symptom score	3 patients were excluded due to violating protocol (H: 1/C:2); 5 patients dropped out due to being lost to follow-up (H:3/L:2); 4 patients dropped out due to other reasons (H: 1/L:2/C:1)	Dizziness, arm itching, urinary tract infection, leukocytosis, abnormal liver function, and rough tongue were observed in 9 patients (H: 4/L:4/C:1)
Min, 2011, China [36]	75/74/73 (T/I/C)	108/114	T: 29.21 ± 7.37 I: 28.21 ± 7.37 C: 30.12 ± 6.01	RCT	T: Bu-tu Xuan-fei decoction; I: Bu-tu Xuan-fei decoction plus compound methoxyphenamine capsules; C: compound methoxyphenamine capsules	Seven	Obvious effective rate;	NS	Stomach bloating was observed in 4 patients in the trial group; dizziness, drowsiness, and fatigue were observed in 12 patients (I:5/C:7)
Huang, 2009, China [37]	30/30	31/29	T: 32.63 ± 9.44 C: 35.20 ± 11.00	RCT; SB	T: Ke-ping decoction; C: loratadine tablets plus dextromethorphan	Seven	Obvious effective rate; cough relief time	No	Drowsiness was observed in 1 patient in the control group

N*: number; T: trial group; C: control group; H: high dosage group; L: low dosage group; I: integrated group; NS: not specified; D: day; DB: double-blind; SB: single-blind.

**Table 2:** TCM principle of CHM and TCM syndrome of patients.

Study ID	CHM intervention	TCM principle of CHM	TCM syndrome of patients
Fujimori et al., 2001 [26]	Extract granules of bakumondo-to (Mai-men-dong decoction in Chinese Pinyin)	Nourishing Yin; descending the upgoing lung-Qi; harmonizing the stomach and lung	Syndrome of lung and stomach Yin deficiency and disharmony between the lung and the stomach
Irifune et al., 2011 [27]	Extract granules of bakumondoto (Mai-men-dong decoction in Chinese Pinyin)	Nourishing Yin; descending the upgoing lung-Qi; harmonizing the stomach and lung	Syndrome of lung and stomach Yin deficiency and disharmony between the lung and the stomach
Qin, 2011 [28]	Er-li decoction	Dispelling wind and dispersing the lung-Qi, resolving phlegm and nourishing the lung-Yin	Syndrome of wind-sputum evil invading the lung
Wang, 2011 [29]	Xuan-fei Zhi-sou decoction	Dispelling wind and dispersing the lung-Qi, resolving phlegm and calming down cough	Syndrome of wind evil invading the lung
Song, 2012 [30]	Qing-fei Zhi-ke decoction	Dispelling wind and dispersing the lung-Qi, eliminating heat and calming down cough	Syndrome of wind-heat evil attacking the lung
Wu et al., 2011 [31]	Shu-feng Xuan-fei decoction	Dispelling wind and dispersing the lung-Qi	Syndrome of wind-evil invading the lung
Zhang et al., 2008 [32]	Su-huang Zhi-ke capsule	Dispelling wind and dispersing the lung-Qi, relieving airway spasm and calming down cough	Syndrome of wind-evil invading the lung
Wu, 2011 [33]	Feng-re decoction; Feng-han decoction	Dispelling wind and dispersing the lung-Qi, expelling cold and relieving exterior; dispelling wind and dispersing the lung-Qi, eliminating heat and relieving exterior	Syndrome of wind-cold evil fettering the lung; syndrome of wind-heat evil attacking the lung
An, 2002 [34]	Zhi-ke Gu-biao decoction	Regulating activity of the lung-Qi and resolving phlegm, tonifying the protective Qi and strengthening exterior	Syndrome of wind evil invading the lung
Jiang, 2013 [35]	Qing-feng Gan-ke granules	Dispelling wind and dispersing the lung-Qi	Syndrome of wind evil invading the lung
Min, 2011 [36]	Bu-tu Xuan-fei decoction	dispelling wind and dispersing the lung-Qi, tonifying the spleen and nourishing the lung-Yin	Syndrome of wind evil invading the lung
Huang, 2009 [37]	Ke-ping decoction	Dispelling wind and dispersing the lung-Qi, relieving sore throat and nourishing the lung-Yin	Syndrome of wind evil invading the lung and Heat evil injuring the lung-Yin

**Table 3:** Risk of bias of included studies.

Study ID	Adequate sequence generation	Random sequence generation (selection bias)	Allocation concealment (selection bias)	Blinding of participants and personnel (performance bias)	Blinding of outcome assessment (detection bias)	Incomplete outcome data (attrition bias)	Selective reporting (reporting bias)	Other bias
Fujimori et al., 2001 [26]	NS	Unclear risk	Unclear risk	Unclear risk	Unclear risk	Low risk	Low risk	High risk
Irifune et al., 2011 [27]	NS	Unclear risk	Unclear risk	High risk	High risk	Low risk	Unclear risk	Unclear risk
Qin, 2011 [28]	NS	Unclear risk	Unclear risk	Unclear risk	Unclear risk	Low risk	Unclear risk	Unclear risk
Wang, 2011 [29]	NS	Unclear risk	Unclear risk	High risk	High risk	Unclear risk	Unclear risk	Unclear risk
Song, 2012 [30]	Yes	Low risk	Unclear risk	Unclear risk	Unclear risk	Unclear risk	Unclear risk	Unclear risk
Wu et al., 2011 [31]	Yes	Low risk	Unclear risk	Unclear risk	Unclear risk	Low risk	Unclear risk	Unclear risk
Zhang et al., 2008 [32]	Yes	Low risk	Low risk	Low risk	Unclear risk	Unclear risk	Low risk	Low risk
Wu, 2011 [33]	Yes	Low risk	Low risk	Low risk	Low risk	Low risk	Low risk	Low risk
An, 2002 [34]	NS	Unclear risk	Unclear risk	High risk	High risk	Unclear risk	Unclear risk	Unclear risk
Jiang, 2013[35]	Yes	Low risk	Low risk	Low risk	Low risk	Low risk	Low risk	Low risk
Min, 2011 [36]	Yes	Low risk	Unclear risk	Unclear risk	Unclear risk	Unclear risk	Unclear risk	Unclear risk
Huang, 2009 [37]	No	High risk	High risk	Unclear risk	Unclear risk	Low risk	Unclear risk	Unclear risk

**Table 4:** Evidence qualities of included studies.

No. of studies	Quality assessment						No. of patients		Effect		Quality	Importance
	Design	Risk of bias	Inconsistency	Indirectness	Imprecision	Other considerations	Chinese herbal medicine	Control	Relative (95% CI)	Absolute		
Cough symptom score (better indicated by lower values)												
5	Randomised trials	Serious^1,2^	No serious inconsistency	No serious indirectness	No serious imprecision	None	216	162	—	Not pooled	⨁⨁⨁◯ Moderate	Critical

Cough relief time (cough relief time) (better indicated by lower values)												
7	Randomised trials	Serious^3^	No serious inconsistency	No serious indirectness	No serious imprecision	None	585	382	—	Not pooled	⨁⨁⨁◯ Moderate	Critical

Cough disappearance time (better indicated by lower values)												
2	Randomised trials	No serious risk of bias	No serious inconsistency	No serious indirectness	No serious imprecision	None	322	128	—	Not pooled	⨁⨁⨁⨁ High	Critical

TCM syndrome clinical effective rate												
9	Randomised trials	Serious^3,4^	No serious inconsistency	No serious indirectness	No serious imprecision	None	687/764 (89.9%)	357/485 (73.6%)	Not pooled	Not pooled	⨁⨁⨁◯ Moderate	Important

Quality-of-life (QoL) score (better indicated by lower values)												
2	Randomised trials	No serious risk of bias	No serious inconsistency	No serious indirectness	No serious imprecision	None	144	89	—	Not pooled	⨁⨁⨁⨁ High	Important

^1^Fujimori et al. stopped the study when the significant differences between two groups were detected, which may lead to an overestimation of intervention in the trial group.

^2^Irifune et al. conducted an open-label trial, which would introduce an influence on subjective patient-report results.

^3^An MC and Wang YF conducted an open-lable study, which would introduce a bias to subjective patient-report results.

^4^Huang MH conducted a quasirandom method, which would introduce selection bias.
